# Editorial: Modeling Epidemics - Why Are Models Wrong?

**DOI:** 10.3389/fpubh.2021.754746

**Published:** 2021-09-09

**Authors:** Theodore Gyle Lewis, Waleed Isa Al Mannai

**Affiliations:** Naval Postgraduate School Alumni, Monterey, CA, United States

**Keywords:** COVID-19, estimating epidemics, predicting cases, total cases, duration of epidemic

## Why Are Models Wrong?

In a paper published April 26, 2020, researchers predicted the number of cases and deaths would exceed 60,000 in the United States by July: “We estimate that through the end of July, there will be 60,308 (34,063–140,381) deaths from COVID-19 in the USA….” (1). The number of deaths exceeded 100,000 by June 6, 2020 and continued to climb surpassing the upper bound on the estimate by late June 2020.

This illustrates one problem with existing models. Estimation turns out to be a hard problem due to missing factors that account for all impacts in the models. “Each model makes different assumptions about properties of the novel coronavirus, such as how infectious it is and the rate at which people die once infected. They also use different types of math behind the scenes to make their projections. And perhaps most importantly, they make different assumptions about the amount of contact we should expect between people in the near future (2).”

We claim that the problem of estimating size and duration extends beyond assumptions about properties of the disease and infectiousness. In addition to the limitations expressed above, population, population density, social network topology, and public sentiment impact the rate and size of the spread of a disease. Mathematical models based solely on properties of the disease are likely to fail, while models based on curve fitting of data and social network theory and public sentiment are more likely to succeed. However, these models failed to accurately predict the size and duration of the COVID-19 pandemic due to a number of technical, social, and public policy issues.

Most models assume a uniformly distributed population with the same levels of immunity or susceptibility to infection, and a relatively immobile population. On the contrary, the modern world violates all of these conditions: populations are clustered, people of different age and economic conditions have different susceptibilities to disease, public opinion as to the dangers of a contagion shift over time, and modern people are extremely mobile.

## Survey of Papers in This Research Topic

Classical models have proven to be inadequate, largely due to a narrow focus on one or more factors rather than on a broad spectrum of factors. Lewis and Al Mannai examines some additional factors that need to be considered, such as population, network structure, and public sentiment. By consideration of factors like population density, size, and duration can vary by an order of magnitude.

An example that illustrates the difficulty of making long-range predictions is found in Reis. The authors performed projection experiments to describe the spread behavior of COVID-19 in four countries Austria, Germany, Italy, and South Korea using mathematical tools such as forward, backward projections, and inverse uncertainty quantification (UQ). They all fail to predict the peaks correctly. The reason is the misestimation of the parameters that describe the time varying transmission rate.

Generally, models can be separated into categories. For example, perhaps the largest category is regression modeling, whereby data are used to fit a logistics function using OLS (Optimal Least Squares). A suitable parameterized function is selected, and its parameters estimated by OLS curve-fitting. In Vicuña the logistic Richards growth curve model is extended by adding a generalized Quasi-Poisson non-linear regression to predict the daily cases of COVID-19 in Chile. Their model predicts short term daily cases pretty well but not in a long term when a second wave occurred.

de Andres compare three approaches across 10 countries: Statistical Heuristic Regression (SHR) model, Susceptible Infected Removed (SIR) model, and Stochastic discrete-time individual based model. They conclude that the SHR model based on a lognormal distribution has an accuracy of 2% for 20 days past the second inflection point in the daily cases while the SIR model had the same accuracy 15 days before.

In Ridder the authors investigate the association between neighborhood socioeconomic deprivation and the persistence of SARS-CoV-2 clusters. By incorporating a broader set of factors they show that the increased risk of infection of disadvantaged individuals is due to the persistence of community transmission.

Alternatively, non-curve-fitting techniques appear to yield comparable results without OLS curve-fitting. In Chen, the authors developed DL (Deep Learning) models to predict the size of the COVID-19 pandemic. They compared the performance of two models: a multivariate model to predict COVID-19 time series for 1, 2, and 3 days ahead, and univariate model to predict new cases, total cases and new deaths. The results show that the univariate model performed better than multivariate model in prediction of COVID-19 for 2 and 3 days ahead, while multivariate model outperformed at 1 day ahead prediction.

Finally, Srivastava reports results of using an agent-based model. The author analyzed effects of the spread of COVID-19 pandemic in a confined community under different lockdown measures, where the agents follow random-walk motion independent of others. Three types of lockdowns are considered: whole population, infected agents, and symptomatic agents. The results after many simulations show that the most effective lockdown is when all infected agents (symptomatic and asymptomatic) are quarantined.

This sampling of alternate models of epidemic spreading illustrates the need for more investigations of epidemic and pandemic models. Models should go beyond traditional curve-fitting and parameter estimating. They need to consider a wider array of factors beyond infection rate and mortality rate. And they need to incorporate the possibility of multiple surges leading to subsequent waves as experienced by the covid-19 pandemic.

## Author Contributions

TL provided introduction and general content. WA provided details on each article. All authors contributed to the article and approved the submitted version.

## Conflict of Interest

The authors declare that the research was conducted in the absence of any commercial or financial relationships that could be construed as a potential conflict of interest.

## Publisher's Note

All claims expressed in this article are solely those of the authors and do not necessarily represent those of their affiliated organizations, or those of the publisher, the editors and the reviewers. Any product that may be evaluated in this article, or claim that may be made by its manufacturer, is not guaranteed or endorsed by the publisher.
